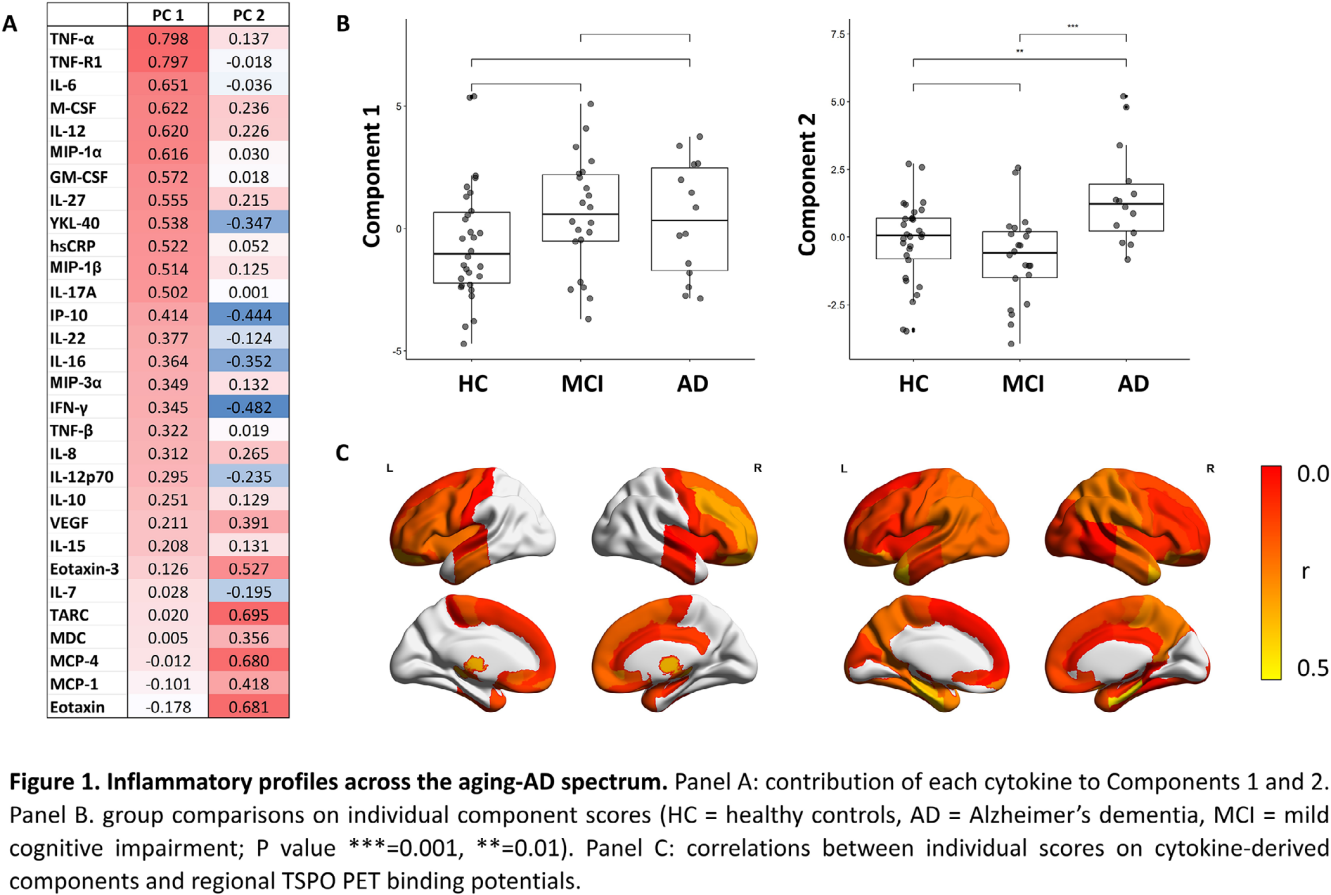# From glia to cytokine signatures across the Alzheimer’s spectrum

**DOI:** 10.1002/alz.092557

**Published:** 2025-01-09

**Authors:** Maura Malpetti, Peter Swann, Leonidas Chouliaras, P Simon Jones, Elijah Mak, W Richard Bevan‐Jones, Li Su, Tim D Fryer, Young T Hong, Franklin I Aigbirhio, John T O'Brien, James B Rowe

**Affiliations:** ^1^ Department of Clinical Neurosciences and Cambridge University Hospitals NHS Trust, University of Cambridge, Cambridge UK; ^2^ University of Cambridge, Cambridge UK; ^3^ University of Sheffield, Sheffield UK

## Abstract

**Background:**

Early neuroinflammation is involved in pathophysiology of Alzheimer’s Disease (AD) and contributes to faster clinical decline. Thus, neuroinflammation has emerged as a promising therapeutic target for dementia. However, a better understanding of the interaction between central and peripheral inflammation in human disease and in vivo biomarkers are required for successful clinical trials. Here we assess inflammatory patterns of serum cytokines and their relationship with central inflammation as quantified by TSPO PET (PK11195) in participants on the aging‐AD spectrum.

**Method:**

Blood samples were obtained from 56 participants, including30 controls, 22 patients with MCI and 14 with AD) Serum cytokines were quantified using the MesoScale Discovery V‐Plex‐Human Cytokine panel. We applied a Principal Component Analysis (PCA) across all participants to identify inflammatory profiles, which were compared across groups with ANOVA and post hoc tests. Individual scores of the resulting components were correlated with regional TSPO PET binding potentials, as index of microglial activation, and cognitive impairment (MMSE scores).

**Result:**

Analyses on 30 identified 2 components (explaining 19.4 % and 10.8% of the variance, respectively). Both components were strongly represented by pro‐inflammatory cytokines (Figure 1A). One‐way analyses of variance on the first component could not detected statistically significant differences across the groups (χ^2^(2)=1.69, p=0.193), while individual scores of Component 2 (χ^2^(2)=8.46, p<0.001) were statistically higher in patients with AD as compared to patients with MCI and to controls (Figure 1B). Correlation analyses with regional TSPO PET values identified positive widespread associations with individual Component 2 scores in frontal, temporal and parietal regions (r > 0.250, p < 0.05; while associations with Component 1 where limited to the anterior frontal/temporal regions (Figure 1C). Individual scores of Component 2 but not Component 1 were significantly associated with worse cognitive performance (lower MMSE; r = ‐0.315, p = 0.01).

**Conclusion:**

Our approach identified proinflammatory blood signatures that relate to central inflammation and cognitive impairment across the aging‐AD spectrum. These findings encourage the combination of TSPO imaging and blood‐based markers to clarify the interaction between peripheral and central inflammation in AD, and to identify promising therapeutic targets.